# Developing Population Health Surveillance Using mHealth in Low-Resource Settings: Qualitative Assessment and Pilot Evaluation

**DOI:** 10.2196/36260

**Published:** 2022-10-14

**Authors:** Natalie C Benda, Sakie Zawtha, Kyrie Anderson, Mohit Manoj Sharma, Phoe Be Lin, Beichotha Zawtha, Ruth Masterson Creber

**Affiliations:** 1 Department of Population Health Sciences Weill Cornell Medicine New York, NY United States; 2 Health and Hope Myanmar Yangon Myanmar

**Keywords:** mobile health, mHealth, low- and middle-income countries, population health surveillance, user-centered design, mobile phone

## Abstract

**Background:**

Population surveillance data are essential for understanding population needs and evaluating health programs. Governmental and nongovernmental organizations in western Myanmar did not previous have means for conducting robust, electronic population health surveillance.

**Objective:**

This study involved developing mobile health (mHealth)–based population health surveillance in a rural, low-resource setting with minimal cellular infrastructure in western Myanmar. This was an early formative study in which our goal was to establish the initial feasibility of conducting mHealth population health surveillance, optimizing procedures, and building capacity for future work.

**Methods:**

We used an iterative design process to develop mHealth-based population health surveillance focused on general demographics (eg, total census, age category, sex, births, and deaths). Interviews were conducted with international consultants (nurse midwives) and local clinicians (nurses and physicians) in Myanmar. Our analytic approach was informed by the Systems Engineering Initiative for Patient Safety work systems model to capture the multilevel user needs for developing health interventions, which was used to create a prototype data collection tool. The prototype was then pilot-tested in 33 villages to establish an initial proof of concept.

**Results:**

We conducted 7 interviews with 5 participants who provided feedback regarding the domains of the work system, including environmental, organizational, sociocultural, technological, informational, and task- and people-based considerations, for adapting an mHealth tool. Environmental considerations included managing limited electricity and internet service. Organizational needs involved developing agreements to work within existing government infrastructure as well as leveraging the communal nature of societies to describe the importance of surveillance data collection and gain buy-in. Linguistic diversity and lack of experience with technology were both cited as people- and technology-based aspects to inform prototype design. The use of mobile tools was also viewed as a means to improve the quality of the data collected and as a feasible option for working in settings with limited internet access. Following the prototype design based on the findings of initial interviews, the mHealth tool was piloted in 33 villages, allowing our team to collect census data from 11,945 people for an initial proof of concept. We also detected areas of potentially missing data, which will need to be further investigated and mitigated in future studies.

**Conclusions:**

Previous studies have not focused heavily on the early stages of developing population health surveillance capacity in low- and middle-income countries. Findings related to key design considerations using a work systems lens may be informative to others developing technology-based solutions in extremely low-resource settings. Future work will involve collecting additional health-related data and further evaluating the quality of the data collected. Our team established an initial proof of concept for using an mHealth tool to collect census-related information in a low-resource, extremely rural, and low-literacy environment.

## Introduction

Reliable and valid population surveillance data are essential for understanding population needs and evaluating health programs [1,2]. Electronic data capture is widely integrated for population health surveillance in high-income countries. Low- and middle-income countries have increasingly demonstrated the feasibility of implementing electronic population health surveillance systems [2-7]. Extremely low–resource settings still face multilevel challenges in establishing sustainable, robust electronic population health surveillance, including sparse or unreliable internet, competing demands of daily life, lack of access to technology devices, low literacy, diverse value structures, and heterogeneous cultural and linguistic design needs [4,7,8].

Mobile health (mHealth) has been presented by the World Health Organization as a means to mitigate some of the aforementioned challenges owing to its relatively low cost, expanding ubiquity throughout the world, and the ability to use cellular networks, which are much more widespread than broadband internet [5,9,10]. Many publications report the evaluation of mHealth interventions in low-resource settings [11,12], but few have described the formative development of population health surveillance systems that support program evaluation [4,5]. In this study, we described the formative development of an mHealth-based tool for conducting population health surveillance in an extremely rural, low-resource, low-literacy environment in western Myanmar. The long-term goal is to support the development of robust infrastructure for population health surveillance in the Chin State of Western Myanmar.

Myanmar presents a unique case related to development of population health surveillance tools, as its borders fully opened to foreign technological influence in the early 2010s; thus, the technological infrastructure lags behind many other low-income countries [9]. As of 2020, Myanmar ranked 147 of 189 on the United Nations Human Development Index [13], and the rural Chin State of Western Myanmar, where this work took place, had the second lowest life expectancy in Myanmar [14]. Residents of this ethnic minority region face barriers to accessing care, including overt racism and an average of 3 days travel to reach a health care facility.

We partnered with a local nongovernmental organization (NGO) that served as the primary provider of health services in the region. The NGO provides health care through local clinics by training community health workers and traditional birth attendants. One of our collaborative goals was to develop a system for mHealth-based population health surveillance to facilitate ongoing needs assessments in villages and evaluation of health programs. Our NGO partner previously collected population surveillance information via paper forms, which presented multiple challenges owing to the time lag from data collection to data entry, as well as the harsh environmental conditions of the remote geography and rainy climate that impede the collection of the data forms in the rainy season. Population health surveillance information helped the NGO track general trends in population (eg, births and deaths), health needs that drive the programs they implement, and evaluate the impact of their programs on population health. Owing to the limitations related to paper-based surveillance, the NGO had never aggregated paper-based data collection in a systematic manner to understand the demographics and needs of their population. mHealth-based electronic data capture of population health surveillance information was proposed given the lower cost of mobile devices, their growing availability in the region, and their flexibility for use in settings with limited internet connectivity [5]. Here, we described formative studies that included interviews and field testing of an mHealth-based population health surveillance data collection tool. The aims of this evaluation were to establish an initial proof of concept and refine processes with the long-term goal-building capacity to use mHealth to provide real-time information regarding census-related information (births and deaths), common health issues addressed by community health workers, and outcomes of local programs (eg, women and infant health education). Our work can be used to inform the needs and best practices to establish a population health surveillance infrastructure in extremely low-resource settings.

## Methods

### Phase One: Semistructured Interviews and Iterative Design

#### Setting

All data were collected via teleconference with stakeholders residing in the United Kingdom or Myanmar by a research team residing in the United States. Interviews with stakeholders in the United Kingdom were conducted via Zoom (Zoom Video Communications, Inc; audio and video), and interviews with stakeholders in Myanmar were conducted via WhatsApp (Meta) or Slack (Slack Technologies; audio only) owing to limitations on internet bandwidth in Myanmar.

#### Description of the Original (Paper-Based) Data Collection Instrument

This study aimed to translate population health surveillance forms previously collected via paper into an mHealth-based tool. Our NGO partners operated in the United Kingdom and Myanmar with clinical experts from the United Kingdom traveling to Myanmar to advise with the design and evaluation of the NGO’s health programs. Paper-based population health surveillance forms were developed by the clinical staff in Myanmar with guidance from United Kingdom–based midwife consultants.

The population health surveillance forms collected census data stratified by age, sex, births per year, and deaths per year. The census data included the total number of men and women in each of the following age categories: 0 to 5 years, 6 to 15 years, 16 to 59 years, and >60 years. Owing to the limitations of the collected paper data, this information had not been previously aggregated; therefore, there was no opportunity for high-level summary and comparison of information over time. The immediate target end users of the tool were the clinical staff from the NGO during outreach trips to villages. In the longer term, the clinical staff will train community health workers living in the villages to use the tool.

#### Study Design and Sample

We first conducted iterative phases of semistructured interviews to select a technology platform and create a prototype of the mHealth-based census form. Our goal was not to achieve theoretical saturation of qualitative data but to iteratively complete formative data collection sufficient for developing a prototype that could be used in a pilot evaluation [15,16]. Stakeholders interviewed included (1) 2 midwife consultants from the United Kingdom and (2) 3 clinical staff from the NGO in Myanmar. Midwives developed paper-based data collection forms used by the NGO. Clinical staff from the NGO included a nurse and 2 physicians with responsibilities such as development and execution of training programs for community health workers, assessment of community health worker competencies, oversight of a local clinic, clinical care at the local clinic, and NGO program evaluation. All participants worked closely with community health workers, who were the eventual end users of the tool. The local NGO staff also had the opportunity to introduce data collection tools to community health workers between rounds of interviews so that they could relay their feedback to the US-based team. Unfortunately, owing to language barriers and internet bandwidth limitations, it was not possible for the US-based team to interview community health workers directly. All interviews were conducted between February and September 2020. The participants were not directly compensated for their time.

#### Data Collection

We completed 3 rounds of interviews with 5 key stakeholders as part of an iterative, formative design process, where smaller numbers of participants (ie, 4-6) were appropriate for needs assessment and design generation [16]. In round 1, we elicited information from all participants about the current processes for paper-based population health surveillance data collection and determined design needs for mHealth-based data collection. On the basis of the findings from the first round of interviews, a design platform (Survey123) was selected (by the NGO staff in the United Kingdom and Myanmar), and initial designs for the mHealth form were created by a member of the US-based team (NCB). Designs were created directly in the survey design, and the data collection platform was selected using a desktop-based program from the vendor.

**Figure 1:**
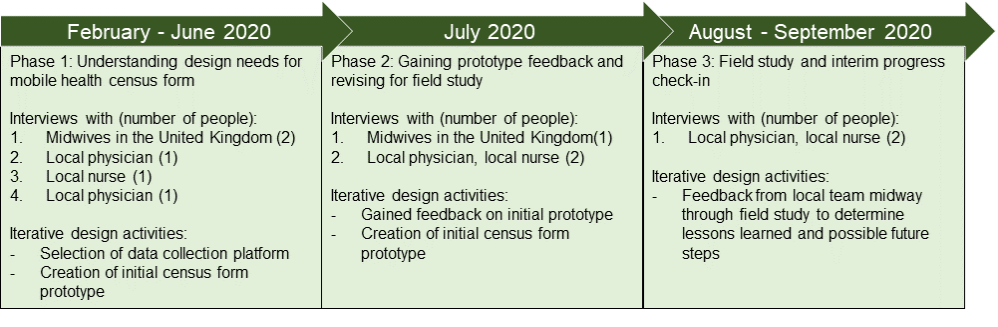
Timeline of interview and iterative design activities.

In round 2, participants viewed the prototype mHealth form and provided their feedback. Round 2 included 2 separate interviews: 1 with a nurse midwife from the United Kingdom and 1 with a nurse and physician in Myanmar. For the staff in Myanmar, forms for second-round interviews were shared via the Survey123 platform before the interview, as live screen sharing was not possible. A final design based on follow-up interviews was created before the field study. An interview was then conducted in the middle of the field studies with the same local physician and nurse from round 2 to understand how the prototype form was suited to the needs of the NGO. Figure 1 presents a timeline of the interviews, prototype design, and feasibility assessment activities.

All interviews focused on current practices, perceived barriers, and facilitators of mHealth data collection. Textbox 1 provides sample questions for both the initial needs assessment interviews and follow-up interviews in which design feedback was collected. Additional probing questions were included in the full interview guide based on the work system components of the Systems Engineering Initiative for Patient Safety (SEIPS), for example, physical environment, organization, people, and task. In line with a semistructured approach, these probes were asked directly in instances when interviewees did not initially discuss each system component.

**Sample interview questions.**

**Needs assessment (round 1) sample questions:**

- Can you tell us about how community health workers fill out the current data collection forms?
- What are the positives of using the paper data collection forms?
- What are some of the challenges for using the paper data collection forms?
- Do you think the mobile phone app could make data collection better? If so, how?
- Do you think the mobile phone could make data collection harder? If so, how?
- Can you tell us more about the community health workers? For example, do most of them read or speak Burmese?
- Do you think the community health workers have used a mobile phone before? A smartphone? Do you think it would be possible for them to learn if we helped train them?
- Is there any more information you think is important for us to know?

**Prototype feedback (rounds 2 and 3) sample questions:**

- We have made a census form. What do you think of it?
  What can we improve?
- What kinds of problems do you think your team may have using the data collection form?
- What new information did you hope to learn from the population surveys?
- How is the information you hope to learn going to help improve (nongovernmental organization name) programs?

All interviews were conducted by a single interviewer (NCB), with other research team members attending and participating as feasible. All interviews were audio recorded and transcribed, excluding an instance where internet connectivity issues prevented the interview from taking place synchronously, so written answers to the interview questions were provided by the interviewee asynchronously.

#### Data Analysis

Our team conducted qualitative interviews using thematic analysis and a constant comparative process [17]. First, a team of 3 coders (NCB, KSA, and MMS) read the transcripts to inductively elicit key themes. The codes were then organized into higher level categories based on work system components highlighted in the SEIPS model, given the complex, intertwined, multilevel constraints involved in a low-resource setting [18-20]. These categories and subcategories were used to develop a codebook consisting of themes, definitions, and exemplars [21]. Once the codebook had been developed, 2 researchers (KSA and MMS) independently coded each transcript, meeting with a third reviewer (NCB) to resolve discrepancies through consensus. After all the transcripts were analyzed, the research team reviewed the results code-by-code to ensure consistency and develop a list of emerging themes. All data were coded using Dedoose (version 8.0.35).

### Phase Two: Pilot Field Study

#### Setting, Study Design, and Sample

The interviews were followed by a field study to establish an initial proof of concept for the use of the mHealth population health surveillance tool in this low-resource setting. Real-time population health surveillance using the census forms developed allowed for the detection of large population changes that required further investigation, as well as allocation of resources to villages based on population. The goal of this pilot study was to establish a proof of concept for using mHealth to collect census-related information and determine potential data quality issues (eg, missingness) that could be optimized through the improvement of data collection tools or training.

The mHealth forms were piloted in villages in the Chin State during outreach visits. The region is characterized by remote, mountainous terrain, so the 33 villages included were a convenience sample located within 1 to 2 days motorbike ride from the NGO’s central headquarters.

#### Data Collection

In the selected villages visited, NGO staff members filled out the population health surveillance forms on mobile devices using a commercially available mapping and survey platform (Survey123). This platform was selected because of its ability to integrate survey-related information with detailed maps necessary to identify the villages served in this remote region. Survey123 is available via app (on Android and Apple) and via web browser. Our partners collected data using tablets and a web browser–based form. Staff members involved in data collection included trained nurses and “area coordinators” (former community health workers who had been promoted into an oversight role). Nurses and area coordinators completed the population health surveillance forms by reviewing paper-based census documentation in the villages (procured from the government and local churches). Paper-based documents often had different age categories, which required the team to add categories from the paper-based forms to suit the categories in the mHealth forms. Nurses and area coordinators then went door-to-door to confirm the census in each sex or age category, number of births that year, and number of deaths. All data were collected between August and September 2020.

#### Data Analysis

Data were downloaded from the Survey123 and summarized descriptively using Microsoft Excel. The proof of concept was assessed by investigating the completeness of the fields included in the form. Summarization included the total number of villages from which data were captured, total persons accounted for, total births, total deaths, and the number of potential missing data fields (Phase Two: Pilot Field Study in the Results section).

### Ethics Approval

This study was approved (20-02021442) by the Weill Cornell Medicine Institutional Review Board and conducted in 2 phases. The first phase involved a series of semistructured interviews and iterative design of an mHealth-based population health surveillance tool with key stakeholders. The second phase involved piloting the tool into a field study.

## Results

### Overview

A total of 7 interviews were conducted with 5 unique participants: 2 midwife consultants and 3 staff members from the NGO. Further description of the interviewees is provided in Table 1. A midwife consultant and 2 staff members were interviewed multiple times.

**Table 1:** Description of participants.

Identifier	Participant description
LocalNurse1	Nurse working for the NGO^a^ in Myanmar, in charge of CHW^b^ training programs
LocalPhysician1	Physician working in Myanmar also serving as the NGO chief operating officer at the time of the interviews
LocalPhysician2	Physician working in Myanmar overseeing the local clinic and medical outreach programs
MidwifeConsultant1	United Kingdom–based midwife that had developed training and data collection for traditional birth attendants affiliated with the NGO
MidwifeConsultant2	United Kingdom–based midwife that had developed training and data collection for traditional birth attendants affiliated with the NGO

^a^NGO: nongovernmental organization.

^b^CHW: community health worker.

### Phase One: Semistructured Interviews

#### Description of Themes

**Figure 2:**
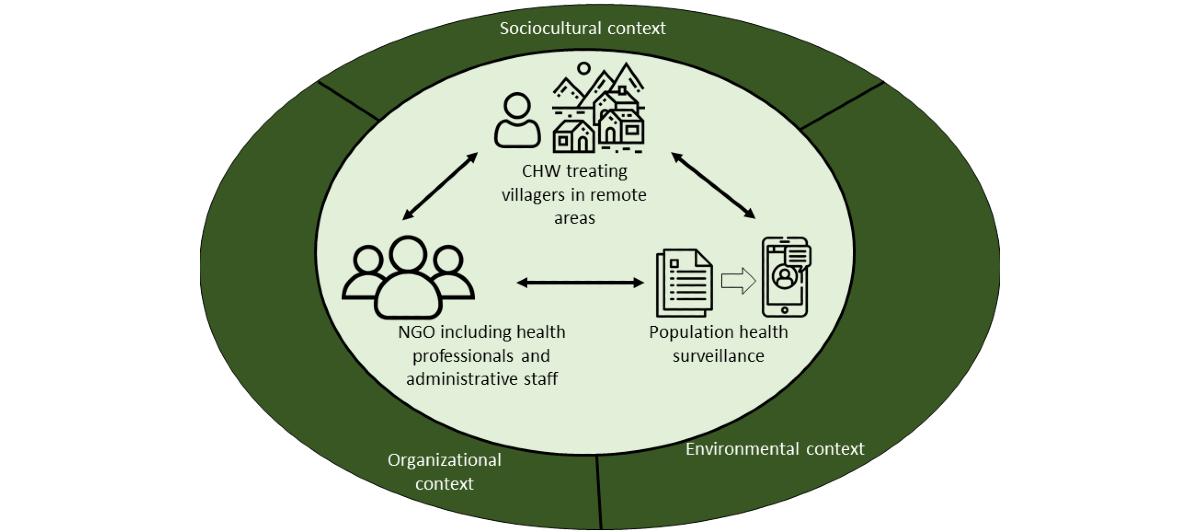
Work system depiction for the current app. CHW: community health worker; NGO: nongovernmental organization.

Key themes pertained to various components of the work system, including the physical environment, organizational concerns, tasks, technology or physical tools, people (user skills and motivation), and information. In line with the traditional SEIPS model, many themes intersected across work system components [20,22]. Figure 2 presents the SEIPS-informed work system for the given app, where community health workers treating patients in villages (people) must communicate information (census and symptoms) to the NGO (people) for the purpose of population health surveillance (task). As this communication previously occurred via paper, the goal of our work was to translate paper data collection tools into an mHealth-based population health surveillance tool to develop capacity for real-time, easy-to-collate data collection. This work was situated in a particular organizational, sociocultural, and physical context that must be considered in the adaptation of new technological tools.

The themes described herein focus on important design considerations for population health surveillance. We have presented the quotes to bolster the description of themes, followed by an identifier indicating the source of the quote. Information included in brackets within the quotes was not explicitly spoken by the participant but provides a clarifying context. Table 1 provides a legend of how identifiers were provided at the end of each quote map for each participant.

#### Physical Environmental Themes

Discussion of environmental themes highlighted the need to consider access to electricity, availability of devices, and internet in low-resource settings when surveillance involved the use of mHealth:

90% [of the villages] they don’t get electricity and they depend on [a] solar system...so whenever they want to charge the phone they would plug in the solar. And not even every household gets solar...only 10 in 30 households get solar.

LocalNurse1

It was also important to consider weather and topography, and how they may impact whether resources can be transported to certain areas during these times of the year:

But in monsoon season, you know we have almost five months rainy season in our country...They rarely...travel. But when they have a better road, a better access to villages to go from here to there by walking or by motorbike, then we ask them to do that. But again, it’s quite difficult.

LocalPhysician1

#### Organizational or Sociocultural Themes

The organizational themes were multilevel, involving the government, cultural norms, and training support from the local organization (ie, our NGO partners). From a governmental perspective, planning was required to effectively manage collaborations with government-funded health entities. Our participants explained that successful integration would involve being aware of the formal and informal structures that exist and using both positively, for example, interfacing with government-funded midwives for maternal and childcare:

But the only problem there is that because they’re [non-NGO affiliated midwives] government-paid...so you'd have to get permission from the government...to use them somehow.

MidwifeConsultant1

Consideration of cultural norms was viewed as a necessity. The region contains predominantly agrarian societies where community health workers provide care to other villagers during their free time when they are not farming. Therefore, it was important to make data collection as simple and efficient as possible to avoid interfering with the competing demands of their daily lives:

They have CHWs, but they are farmers, and they are just volunteer people...so they have to work for their living as well. At the same time, they have to serve the community, so it is really challenging for them.

LocalPhysician2

The cohesive nature of the villages was seen as a potential motivator for buy-in with data collection if the NGO trainers could illustrate how collecting population health surveillance information impacted the health of the larger community (People-based considerations). Finally, providing in-person training and additional ongoing support, possibly remotely, was also a necessary consideration.

#### Task-Related Themes

Participants described the need to support key tasks related to population health surveillance, including detection of problematic public health patterns, measurement of program progress or effectiveness, and promotion of safe health practices. The detection of public health problems would allow for better allocation of resources (eg, medicines and first aid) and additional training to address emerging problems, such as the COVID-19 pandemic:

Data were very helpful especially during Covid-19 outreach. It was easy for us to choose the villages based on the data form and was easy for preparation of materials based on the population they provided. As for me data is most useful in preparation of medicines and materials to distribute to the active villages. I can prepare medicines and materials based on the number of active CHWs and population of the villages.

LocalNurse1

#### Technology Themes

Key desired specifications related to the technological tools included: low cost, inclusion of data security protection (eg, password protection and remote wipe capabilities), ability to transmit information to cloud-based databases, ease of use, flexibility (to edit forms as needed), and capabilities to work in the given environment (eg, protection during the rainy season and store-and-forward capabilities for areas with limited cell service):

Yes, I think using [a] mobile phone app for data collection will be a perfect choice for the...CHWs...It is easy to carry and once it is filled there is no need to worry about the loss of information. It will be done at once so time saving...The outcome of data will be more accurate. It is more simple to understand on mobile.

LocalNurse1

#### Person-Related Themes

Person-based design needs pertained to user skills and motivations. Different user needs or skills to accommodate multiple languages, have limited literacy, and little to no experience with mobile technologies:

One of our huge obstacles has been the translation...they really genuinely don't read each other's dialects, don't always follow each other's dialects, and so on. So, again, it's not just illiteracy, it's overcoming language barriers that would make some sort of pictorial and touchscreen thing easier.

MidwifeConsultant2

[The] mobile phone thing is very new. Last year (2019) we have got this telephone tower and people start[ed] using phones.

LocalPhysician1

Another person-based theme involved understanding and leveraging potential motivators for community health work and data collection. Avenues for motivation overlapped with other work system components such as reimbursement or payment provided by the local NGO, respect for other villagers and desire to help, excitement related to new technologies (ie, mobile phones), and personal altruism. It was also viewed that connecting the data collection activities to the larger purpose of the different organizations (both the NGO and village) would serve as a motivating factor:

We also have to explain why we make them use this app...because we would like [to know about] their village and how the patients are being taken care of by them, and then it’ll be...a strong connection between the community and us [the NGO].

LocalPhysician2

#### Information-Related Themes

Information-related themes touched on data quality and data structure. In general, data quality was perceived to be higher when using the mHealth tool over paper forms as it eliminated issues with reading handwriting. Structured data entry was also viewed as helpful in reducing issues related to limited literacy, although local team members wanted unstructured data entry to account for atypical cases:

Some of the CHWs are not so educated so they do not know the exact words to fill sometimes.

LocalNurse1

Actually, here “others” is something that involves nothing that we have here...If it's out of this topic and you see something other than this or that, please describe it. That's how we taught them.

LocalPhysician2

#### Iterative Design

**Figure 3:**
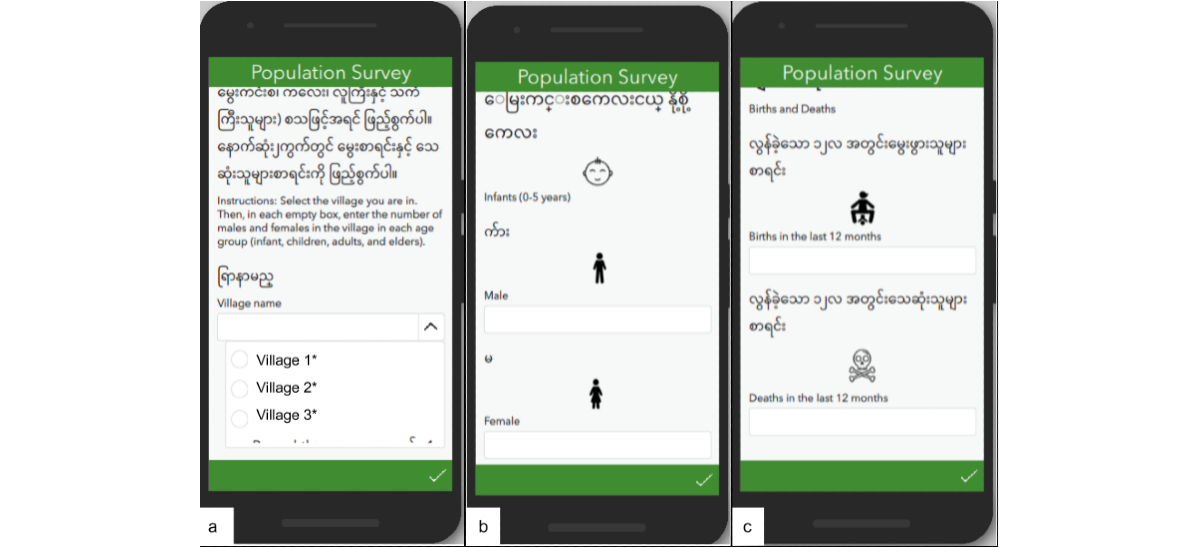
Population health surveillance form for collecting village census information. Village names have been redacted but included the village name in Burmese characters as well as the unique, numeric identifier.

Figure 3 shows key design features of the population health surveillance form developed for census tracking based on the iterative interviews with key stakeholders previously described. Figure 3A shows instructions provided in Burmese (and English for comparison purposes). Users were first able to search the village for which they are completing the survey. The form entry contained both Burmese text and icon-based representation of the information to be entered (Figures 3B and 3C). In addition to the visual design features, Survey123 allowed for local device data storage when there was insufficient internet service but would then automatically upload the information to a secure cloud server once the device had service again, thus mitigating previously described concerns related to internet connectivity. Table 2 provides a complete list of updates made to the prototype in advance of the pilot study on the basis of the interviews.

**Table 2:** Approaches implemented for the design considerations derived from interview themes. Work system domain.

Particulars and design considerations			Approach implemented
**Environmental**			
	Lack of electricity for charging devices	Included solar charged batteries in budget to supplement existing solar-based systems	
	Mountainous topography, transportation challenges owing to weather	Choose a mapping software to find villages in need	
**Organizational**			
	Planning of government vs NGO^a^ ownership of materials	Developed a plan where NGO would purchase and own devices, developing memorandum of understanding with the government	
	Need to balance data collection with demands of daily life	Created simple-form design so data collection is as efficient as possible	
**Task to support**			
	Detection of problematic public health patterns	Chose a mapping platform, which allows geographic tracking and data summarization	
	Program measurement	Chose a mapping platform, which allows geographic tracking and data summarization	
**Technology**			
	Low cost	Found a program usable on Android (ie, for mapping and survey) offering nonprofit pricing	
	Flexibility to edit forms	Used a web platform for NGO staff members to edit forms easily	
	Work in the given environment	Purchased waterproof cases for devices	
**People**			
	Easy to learn and use	Leveraged an iterative participatory design process to create data collection forms	
	Multiple languages	Included Burmese text in forms	
	Limited literacy	Included icons in forms	
	Data structure (unstructured vs structured)	Made most fields structured Included open-ended text boxes for additional notes not conforming to structured data collection elements	

^a^NGO: nongovernmental organization.

### Phase Two: Pilot Field Study

**Figure 4:**
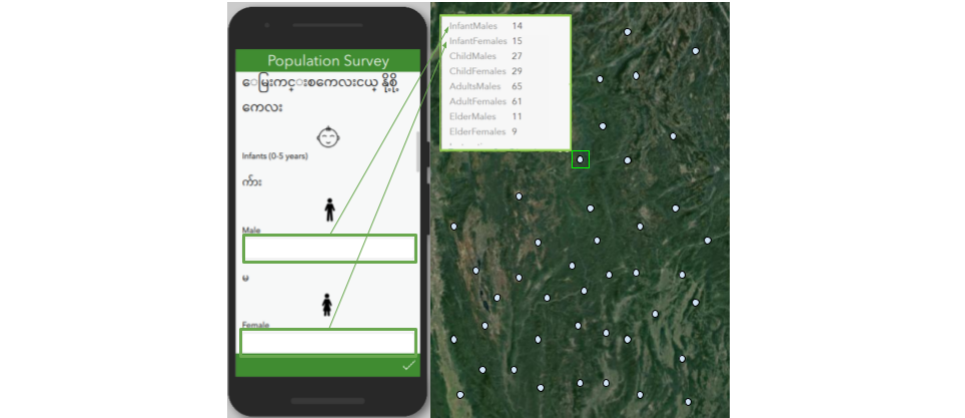
Demonstration of how the survey platform from Survey123 (left) integrates with the mapping platform (right).

The prototype displayed in Figure 3 was used to capture population information in 33 villages by the NGO staff, including nurses and area coordinators who oversaw the community health worker training program. The prototype was created using Survey123 Connect for ArcGIS (version 3.9.120; Esri) Figure 4 illustrates how the census information collected using the survey app is integrated with the mapping app to allow users to view specific population statistics and detect patterns in population growth or decline. Data collection in 33 villages allowed our NGO partners to account for 11,945 people in prespecified demographic groups based on age and sex. In addition, they captured 205 births and 27 deaths across the villages. There were potential missing data from 8 and 17 villages regarding birth and death counts, respectively, but there were no missing data indicated for the general census information (ie, census of villagers in age and sex groups). The missing data may indicate that birth- and death-related information is more challenging to determine or that the users simply did not know that they should enter the number “0” to indicate none instead of leaving the field blank.

Feedback from the study in the field (third round interviews) provided a proof of concept for electronic (mHealth-based) population health surveillance data collection and underscored the need for store-and-forward capabilities for villages that may not have sufficient cellular service for real-time data upload. For example, in one of the follow-up interviews, an NGO-employed nurse described the following:

So far, it's very good and it is very easy to use...I think compared to the paper form...I prefer these Survey123 survey data forms.

LocalNurse1

## Discussion

### Principal Findings

Our formative, iterative design approach allowed us to develop an mHealth tool for population health surveillance in an extremely rural, low-resource, limited-literacy environment with minimal cellular infrastructure in western Myanmar. We first summarized our results and illustrated how they were translated into concrete next steps, as the implementation of our findings may be informative for others conducting similar work. We then described our findings in the context of the work system model. Finally, we discussed the overlap of our findings with those of other studies and highlighted how our results extend currently published knowledge.

Key design needs were organized based on environmental, organizational, sociocultural, technological, and task-, people-, and information-based constructs. Many of the design considerations derived from the interviews were addressed before the field study (Table 2). Our field study provided an initial proof of concept by piloting the mHealth tool for in situ data collection in 33 villages. However, additional considerations were not necessary or feasible to implement at the time of the pilot study. For example, training considerations for community health workers were not needed for the pilot study, as it was conducted by the NGO staff. Table 3 presents further design considerations from our interviews, which were operationalized in later ongoing studies.

As shown in Tables 2 and 3, many of the considerations did not involve the design of the mHealth tool but instead pertained to the design of processes, training, and selection of technologies. In addition, some considerations were addressed through multiple solutions, and others addressed multiple considerations. Although example design considerations are listed under the primary work system domain, the examples also highlight the overlap of the domains. For example, building logic checks in the data collection form (information) may also mitigate issues related to limited literacy and ease of use (people). However, error messages may need to be conveyed in both written and audio formats to those with limited literacy. Additionally, built-in logic checks must be facilitated by the chosen technology.

Table 3 describes the steps implemented in subsequent ongoing work. We further explored the reasons for data missingness. In addition, our ongoing work extended the described mHealth tool to allow for collection of noncommunicable diseases and maternal or child health information to better understand patterns in health needs within the villages that may also explain changes in census. Our approach for evaluating data plausibility described in Table 3 will also provide quantitative metrics to evaluate data quality and make further improvements. The ongoing refinement of population health surveillance tools is underway in preparation for future clinical trials to evaluate maternal health interventions.

Previous studies regarding the integration of electronic health surveillance and mHealth integration into low-resource settings are similar to ours, but others may not yet be applicable in particularly underdeveloped settings. For example, McIntosh et al [4] also noted the importance of store-and-forward technologies in areas where internet connectivity may be sparse. The weather and topography issues encountered in our study, which may limit travel to allow for regular data uploads, present an additional complication and consideration for our study. Berry et al [23] described an approach for population health surveillance in Bangladesh using random digit dialing instead of the door-to-door approach used in our work. Other studies in south Asia and Sub-Saharan Africa have also demonstrated the feasibility of using SMS text messaging for basic data capture and sending reminders to improve adherence to maternal and child health regimens [24-26]. However, as noted by one of our participants, cell phone towers have only recently been constructed in many villages (as of 2019); therefore, cell phones are not yet a ubiquitous, equitable means for communicating with the population of the Chin State. Our results may be particularly helpful for regions with less developed cellular infrastructure as well as those facing climate- and geography-related challenges.

**Table 3:** Considerations derived from interviews implemented in subsequent efforts.

Work system domain and design considerations		Implemented approach
Environmental considerations derived implemented in pilot study		Considerations derived implemented in pilot study
Ongoing organizational training and support		Developed videos for data collection forms to providing ongoing training
**Task to support**		
	Considerations derived implemented in pilot study	Considerations derived implemented in pilot study
	Secure data collection and storage	Obtained software that may be locked (with a passcode) and wiped remotely
	Ability to transmit information to database	Purchased cords for data transfer so data could be stored and uploaded later in villages with poor cell service
**People**		
	Easy to learn and use	Iterative participatory design
	Multiple languages	Added text or audio from additional local languages (eg, Mara)
	Limited mobile device experience	Incorporated mobile device basics into data collection training
	Motivating data collection	Connected data collection to larger purpose of the work in training Providing needed supplies to community health workers Created a data plausibility review process by NGO^a^ staff

^a^NGO: nongovernmental organization.

### Limitations

Our study has limitations related to (1) sample size for the iterative design process, (2) field study, (3) transferability of findings, (4) ongoing COVID-19 pandemic, and (5) military coup in the region. First, our interviews involved 5 participants and 7 total interviews, which are smaller than sample sizes recommended in some qualitative guidelines but appropriate for our approach—an iterative, formative design process [15,16,27]. Our goal was not to meet qualitative saturation but to pragmatically sample to gain enough information for our iterative design process. Previous studies on technology design have indicated that ≥5 participants may be sufficient for early-stage formative design [16,28]. Our initial studies focused on the clinical staff of the NGO, and future studies will involve community health workers who will also eventually use mHealth data collection forms to provide more timely information from each village to the NGO’s central headquarters. Second, the field study did not formally evaluate feasibility (eg, via survey) or data quality; rather, it was a proof of concept. We plan to include ratings of data plausibility by trained staff members and retraining as needed to record implausible values in future efforts, and to assess the timeliness of data upload to detect the effect of connectivity issues in certain areas [29]. Third, data were collected from a single NGO in a specific Myanmar region. Although we sought to determine general design considerations for extremely low-resource settings, it is unclear whether our results will be transferable to other settings. Fourth, we originally planned to conduct formative interviews and field assessments with members of our US-based research team traveling to Myanmar for in-person data collection. Owing to safety precautions and travel restrictions related to the COVID-19 pandemic, this was not possible at the time of the study. Finally, on February 1, 2021, the democratically elected government of Myanmar was overthrown by a military coup and the country was in a state of emergency; as such, the NGO operations were suspended.

### Conclusions

We conducted formative, qualitative interviews with key stakeholders to develop an mHealth-based system for population health surveillance in a low-resource setting in Myanmar. Design considerations involved various work system domains including physical environment, organization-, task-, technology-, people-, and information-related issues. Members of our NGO partners were able to use the mHealth-based population health surveillance tool to establish an initial proof of concept in a feasibility study of 33 villages. Future work will involve evaluating the plausibility of the data collected and expanding data collection beyond simple census information to disease and maternal or child health outcome information. The February 2021 military coup in Myanmar presented a difficult political environment for the employment of this approach in our continued work that will also need to be addressed, moving forward regarding safety and security reasons.

## Abbreviations

mHealth: mobile health
NGO: nongovernmental organization
SEIPS: Systems Engineering Initiative for Patient Safety
